# Is Growth Restriction in Twin Pregnancies a Double Challenge? – A Narrative Review

**DOI:** 10.34763/jmotherandchild.20202404.d-20-00016

**Published:** 2021-07-06

**Authors:** Dagmara Filipecka-Tyczka, Grzegorz Jakiel, Anna Kajdy, Michał Rabijewski

**Affiliations:** 1 Department of Reproductive Health, Centre of Postgraduate Medical Education, Warsaw, Poland; 2 Centre of Postgraduate Medical Education, 1st Obstetrics and Gynecology Clinic, Warsaw, Poland

**Keywords:** definitions, foetal growth restriction, hypotrophy, management, twin pregnancies, twins

## Abstract

**Background:**

Foetal growth restriction (FGR) complicates about 25–47% of twin pregnancies. One or both foetuses can be restricted. Pregnancies with discordant growth of foetuses are associated with a sevenfold increased risk of neonatal morbidity.

**Materials and methods:**

This is a narrative or traditional literature review. A literature search was performed to present a comprehensive, critical and objective analysis of the current knowledge on growth restriction in twin pregnancies.

**Results:**

The definitions of FGR in twin pregnancies and selective FGR (sFGR) differ between international societies. In 2019, the Delphi procedure aimed to unify the definitions of sFGR in twin pregnancies. Several growth charts for twins have been published. However, most societies recommend singleton growth charts as better in detecting hypoxic complications of FGR in twin pregnancies. Discordant growth in twins results from placental insufficiency, congenital anomalies, chromosomal aberrations and TORCH infections.

**Conclusions:**

Definitions and management of sFGR depend on chorionicity. The management aims to protect the properly growing foetus from ischemic complications or in utero death. In most cases, expectant management, strict surveillance and preterm labour are the methods of choice. Due to the co-existence of properly growing and small foetuses in one uterus, determining the appropriate time for delivery is challenging. In the case of preterm labour, even late preterm, antenatal corticosteroid therapy (ACT) in FGR twin pregnancies is beneficial because it decreases neonatal morbidity.

## Introduction

Twin pregnancies have an increased risk of mortality and morbidity. Many pregnancy complications are more common in multifoetal gestations than in singleton gestations. Foetal growth restriction (FGR) is one of the most frequent pathologies. It complicates about 25–47% of twin pregnancies^1^, ^2^ and only 8% of singletons.^3^, ^4^ It occurs in 11–24% of dichorionic (DC) twin pregnancies and 20–45% of monochorionic (MC) twin pregnancies.^5^ One or both foetuses can be restricted. Pregnancies with discordant growth of foetuses or with one growth-restricted foetus are associated with a sevenfold increased risk of neonatal morbidity.^6^

In cases of selective FGR (sFGR), pregnancy management is a challenge. Preterm delivery may increase the chance of survival of a severely restricted twin. However, at the same time, it exposes the properly growing foetus to complications of prematurity and the risk of neonatal death. Expectant management may increase the chances of the appropriately growing foetus in a DC pair due to the avoidance of extremally prematurity. However, the problem becomes more complicated in MC pregnancies complicated with sFGR. In MC twin pregnancies, there are arteriovenous and venous-arterial anastomoses in the shared placental plate. Through anastomosis, the blood transfuses between the foetuses. The intrauterine death (IUD) of one of the foetuses in MC impacts the state of the co-twin. A properly growing foetus may bleed to a dead twin. In this scenario, the IUD of one foetus may lead to hypovolemia of the survivor and severe injury or in utero death.^7^, ^8^, ^9^

## Methods

This is a narrative or traditional literature review. A literature search was performed to present a comprehensive, critical and objective analysis of the current knowledge on growth restriction in twin pregnancies.

The search strategy in PubMed and SCOPUS was based on Medical Subject Headings (MeSH) terms. We searched for keywords appearing in the works in this field in the MeSH Database which allowed us to construct the query: (“Fetal Growth Retardation” [Mesh]) AND (“Pregnancy, Twin” [Mesh] OR “Twins” [Mesh]). Besides, we looked for the existing recommendations of national or international scientific gynaecology and obstetrics societies in the field of FGR in twins. Subsequently, we removed duplicates and verified the abstracts to meet the inclusion and exclusion criteria. Inclusion criteria were original papers, systematic reviews and scientific societies’ recommendations, whereas the exclusion criteria were reports on single pregnancies or more than two foetuses. The search included the articles in English and Polish languages only, published in the past 15 years (2005– 2020).

We reread the publications’ full texts. The main topics highlighted are as follows: definition of FGR, growth charts, aetiology of FGR/sFGR, follow-up, management and antenatal corticosteroid therapy (ACT) in FGR twin pregnancies. The analysis and summary of the results of the work were carried out according to the abovementioned topics.

## Results

### Definition of sFGR

The definitions of FGR in twin pregnancies and sFGR differ between international societies.

Discordant foetal growth in multifoetal gestations is defined as a 15–30% difference in the estimated foetal weight (EFW) between foetuses. This growth discordance ratio is calculated by subtracting the weight of the smaller foetus from that of the larger one, dividing it by the weight of the larger foetus and multiplying by 100%,^7^, ^10^ as shown below:


$$\text{EFW discordance}\;\;=\frac{\left(EFW\text{ large foetus }-EFW\text{ small foetus }\right)}{\text{ EFW large foetus }}\times 100\text{\%}$$

The National Institute for Health and Care Excellence (NICE, 2019), the International Society of Ultrasound in Obstetrics and Gynecology (ISOUG) and the American College of Radiology (ACR) guideline for FGR in twins define sFGR as discordance of EFW 25% and above or EFW of one foetus below the 10^th^ centile for gestational age (GA).^6^, ^7^, ^11^ The same definition of sFGR is used by the International Federation of Gynecology and Obstetrics (FIGO), but it recommends twin-specific growth charts to avoid overdiagnosis.^12^ The American College of Gynecologists (ACOG) defines sFGR as one foetus having EFW below the 10^th^ centile. However, according to ACOG, EFW discordance is defined as exceeding 20%.^13^ The Royal Australian and New Zealand College of Obstetricians and Gynaecologists (RANZOG) published a recommendation on MC twins but it does not define FGR.^14^ The Society of Obstetricians and Gynaecologists of Canada (SOGC) states that increased foetal surveillance is indicated when EFW or abdominal circumference (AC) of one or both foetuses is below the 10^th^ centile or when growth disproportion is identified. They do not specify the type of growth curves (singleton or twin).^10^ The Polish Society of Gynecologists and Obstetricians (PSGO) has not yet published recommendations on twin management. See Table 1 for a comparison of the presented definitions.

**Table 1:** sFGR definition in twins among societies.

Society	Date of publication	Definition of sFGR	Growth charts	Other twins recommendation
NICE	2019	≥25% EFW discordance and EFW of one foetus <10^th^ centile for GA	Undefined	
ACOG	2019	One foetus has EFW <10^th^ centile and disproportion between EFW >20%	Undefined	
ISOUG	2016	One foetus has EFW <10^th^ centile and the intertwin weight discordance >25%	Singleton	
RAN-ZOG	2017	Undefined	Undefined	Recommendation about MC twins but without definitions of FGR
SOGC	2017	AC and/or EFW of one or both twins are <10^th^ centile or when growth discordance is identified	Singleton	
ACR	2017	One foetus EFW <10^th^ centile and the intertwin EFW discordance >25%	Singleton	
FIGO	2019	One foetus EFW <10^th^ centile and the intertwin EFW discordance >25%	Twin	
Delphi consensus	2019	Separates MC and DC twins, EFW <3^rd^ centile *or contributory factors*	Singleton	

AC, abdominal circumference; ACOG, American College of Gynecologists; ACR, American College of Radiology; DC, dichorionic; EFW, estimated foetal weight; GA, gestational age; FIGO, International Federation of Gynecology and Obstetrics; ISOUG, International Society of Ultrasound in Obstetrics and Gynecology; MC, monochorionic; NICE, National Institute for Health and Care Excellence; RANZOG, Royal Australian and New Zealand College of Obstetricians and Gynaecologists; sFGR, selective FGR; SOGC, Society of Obstetricians and Gynaecologists of Canada.

In 2019, a Delphi procedure defined sFGR in twin pregnancies. Results were published to unify definitions and help future researchers to compare the results of studies. According to the published definition, all twin foetuses with EFW below the 3^rd^ centile are growth-restricted. If EFW is below the 10^th^ centile but above the 3^rd^ centile, sFGR is defined according to chorionicity. In MC twins, the following two out of four criteria have to be met to recognise sFGR: (1) EFW of one foetus <10^th^ centile, (2) AC of one foetus <10^th^ centile, (3) discordant ratio ≥25% and (4) umbilical artery pulsatility index (UAPI) of the smaller foetus > 95^th^ centile. In DC, to detect sFGR, the following two out of three circumstances have to be met: (1) EFW of one foetus <10^th^ centile, (2) discordant ratio ≥25% and (3) UAPI of the smaller foetus >95^th^ centile^7^, ^15^ (see Table 2). The ISUOG recommends calculating EFW on the combination of head, abdomen and femur measurements.^7^

**Table 2:** sFGR definition by expert consensus: A Delphi procedure.

Definition establish in Delphi procedure	
DC twins	MC twins
EFW <3^rd^ centile or	
EFW of one foetus <10^th^ centile	EFW of one foetus <10^th^ centile
AC is not taken into account	AC of one foetus <10^th^ centile
The disproportion between foetal weight ≥25%	The disproportion between foetal weight ≥25%
UAPI of smaller foetus >95^th^ centile	UAPI of smaller foetus >95^th^ centile
2/3 have to be present to recognise sFGR	2/4 have to be present to recognise sFGR

AC, abdominal circumference; DC, dichorionic; EFW, estimated foetal weight; MC, monochorionic; sFGR, selective FGR; UAPI, umbilical artery pulsatility index.

### Growth charts

Several growth charts for twins have been described. Twin growth in the third trimester is slower when compared with singletons. In the STORK study and the Shivkumar group, data disproportion between twins and singletons can be observed from 28 weeks of gestation in MC pregnancies and 32 weeks in DC pregnancies.^16^, ^17^ At the same time, numerous experts are concerned that the reduced growth of twins results from placental pathology, and the usage of twins’ growth curves may delay the diagnosis of abnormal growth in multiple pregnancies. The Delphi definition and most societies recommend to use singleton growth charts to assess foetal growth in twin pregnancies.^7^, ^10^, ^11^, ^15^ The comparison of growth charts used by international societies is shown in Table 1.

### Aetiology of FGR/sFGR

The leading cause of growth restriction in multiple pregnancies is placental insufficiency. The uterine vascular bed is inefficient in supplying the double mass placenta with oxygen and nutrients. Other causes of FGR, similar to singletons, are chromosomal abnormalities, congenital anomalies or infections.

In MC twin pregnancies, the primary and unique cause of sFGR is unequal sharing of the placental bed, but it is still possible of the existing mentioned causes.

After the diagnosis of FGR, ISUOG recommends the reassessment of anatomy, exclusion of TORCH infections (cytomegaly, toxoplasmosis, herpes infection, syphilis and rubella) and amniocentesis if chromosomal aberrations are suspected.^7^, ^18^

### Follow-up

The surveillance of FGR in twins includes serial biometrical measurements and Doppler assessment. Different follow-up and management are necessary due to the different placenta structure and additional risks for MC than DC pregnancies.

#### DC twins

In DC twins, ISUOG recommends sequential ultrasound examination for every 4 weeks from 24 weeks of gestation, Doppler evaluation of middle cerebral artery (MCA), umbilical artery (UA), and ductus venosus (DV) flow and biophysical profile scores as in growth-restricted singletons.^7^, ^18^, ^19^

As in singletons proposed by Figueras, there are four types of FGR recognised according to abnormal Doppler assessment/ or abnormal cardiotocography (CTG). Type I is characterised by PI UA above 95^th^ centile and positive end-diastolic flow (EDF) or cerebroplacental ratio below CPR 5^th^ centile. This stage is connected with a good prognosis. Type II features absent EDF (AEDF), which is a poor prognostic indicator. Type III features reverse EDF (REDF) and/or ductus venosus pulsatility index (DV PI) above 95^th^ centile. Type IV is when reversed DV a wave or abnormal CTG is observed. Abnormal DV wave is the strongest parameter to predict the short-term risk of foetal demise.^19^

#### MC twins

In MC twin pregnancies, the recommended surveillance of EFW and Doppler begins at 16 weeks of gestation.^7^ MCA Doppler may be disturbed due to anastomosis in a single placenta with a shared vascular net. Therefore, Gratacos et al.^20^ proposed a classification of sFGR in MC twins based on EDF in UA Doppler. Three types of growth restrictions are defined (see Figure S1 in Supplementary Material). Type I has a positive EDF and a good prognosis. Type II features absent/ reverse EDF (AREDF), which is a poor prognostic indicator. Type III is unique for MC pregnancies and presents with intermittent AREDF (iAREDF). In a shared placenta, large-diameter anastomoses allow cyclical compensatory flow between foetuses. The prognosis in Type III is unpredictable.

**Figure S1:**
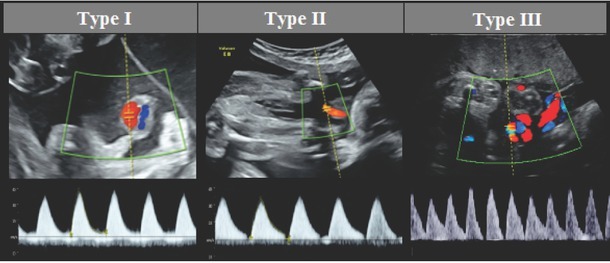
Ultrasound images of umbilical flow according to the classification of FGR in MC twins by Gratacós et al.^20^ FGR, foetal growth restriction; MC, monochorionic.

In all reported cases with iAREDF, MCA and DV PIs were normal before the restricted twin’s IUD.

The prognosis in sFGR in MC twins depends on the severity of Doppler changes. In Type I, the overall risk of IUD is <4%. In Types II and III, the risk of IUD is similar (16% and 12%, respectively). However, the risk of sudden death of the smaller foetus is unpredictable in Type III, even if the condition seems to be stable by ultrasound.^20^, ^21^

### Management

According to Khalil et al., there are limited data to guide the management of twins affected by sFGR.

#### DC twins

In DC twin pregnancies complicated by FGR, many societies recommend a biweekly weekly assessment of foetal UA and MCA Doppler and amniotic fluid volume^7^, ^11^ (see Table S1 in Supplementary Material).

**Table S1:** Assessment of hypertrophic twin pregnancies depending on chorionicity.

sFGR twins	Color Doppler	Foetal growth assessment
DC	Biweekly or more frequent	Biweekly
MC	Once a week or more frequent	

DC, dichorionic; MC, monochorionic; sFGR, selective FGR.

There is little research about the management of FGR/sFGR in DC twin pregnancies, but good clinical practice recommends similar care to propose for singletons complicated by FGR. The main difference is the time of delivery. The aim is to continue the pregnancy as long as possible in the properly grown co-twin’s interests. Due to this, the delivery time ought to be after 30–32 weeks of pregnancy if possible. In certain severe early restricted cases, complicated by severe preeclampsia, the selective reduction is possible in DC twin pregnancies. The sFGR twin’s selective feticide may resolve pre-eclampsia and extend pregnancy duration to obtain better obstetric outcomes in a properly growing foetus.^7^, ^18^

In Poland, selective termination of smaller twin after 22 weeks of gestation is not permitted by law.

#### MC twins

In MC twins, a weekly assessment of foetal UA and MCA Doppler and amniotic fluid volume is recommended^7^, ^11^ (see Table S1 in Supplementary Material).

Sukhwani et al.^22^ suggest expectant management in Type I sFGR in MC twins due to a good prognosis. In Types II and III, the clinical decision is a challenge and requires individualised care. Management depends on the severity of sFGR, UA Doppler changes, GA at diagnosis, technical capabilities and parents’ preferences.

As Miyadahira et al.^23^ reported in 2017, three management options are available for Types II and III sFGR MC twins: expectant management, selective laser photocoagulation of placental connecting vessels (SLPCV) or cord occlusion of the smaller twin. In several countries, cord occlusion in cases of sFGR is not allowed after 22 weeks of pregnancy. Brasil group proved that SLPCV in severe cases with abnormal DV flow before 26 weeks gestation improved perinatal outcomes. A more decisive option is selective termination of the small twin in cases with abnormal DV Doppler (absent or reversed a-wave) before 26 weeks of pregnancy. The procedure aims to protect the normally grown foetus from the consequences of the smaller twin’s death. In cases with abnormal a-wave in DV Doppler above 26 weeks, delivery is recommended.^7^, ^24^

Conservative management is an option in both types of twin pregnancies. Surveillance until the deterioration of the smaller twin with preterm labour is the last resort treatment. However, according to Townsend, pregnancy should be continued as long as possible in the properly grown foetus.^18^

In MC twins before 26 weeks of gestation, fetoscopic interventions are recommended in sFGR Types II and III due to a high risk of the properly grown foetus’s morbidity and mortality. In severe cases, the recommended procedure is cord occlusion by radiofrequency ablation (RFA) or bipolar coagulation. A more complicated procedure is SLPCV, but it also gives a chance to the smaller twin.

Table 3 presents the GAs for delivery of sFGR, recommended by experts, according to Doppler assessment. DC, MC twins, and singletons are compared.^7^, ^18^, ^19^, ^20^, ^24^

**Table 3:** Recommended GA at delivery in stable twins pregnancies complicated by sFGR according to Doppler assessment compared with FGR singleton pregnancies.

		sFGR Type		
Chorionicity	I	II	III	IV
**DC**	UAPI >95^th^ centile CPR <5^th^ centile	AEDF	REDF, DV PI >95^th^ centile	DV a wave reversed
**MC**	**I**	**II**	**III**	
	UAPI >95^th^	AREDF	iAREDF	DV a wave reversed (it is not stage IV in MC)
Recommended GA at delivery (weeks)				
**DC**	34–36	30–32	30–32	>26 <26 expectant management or selective termination^*^
**MC**	34–36	30–32	30–32	>26 <26 SLPCV, selective termination (RFA, cord occlusion)
**Singletons**	37	34	30	26 <26 expectant management

Data reproduced from Figueras and Gratacós,^19^ Gratacós et al.,^20^ ISUOG guideline and Khalil et al.^7^ AEDF, absent EDF; AREDF, absent/reverse EDF; DC, dichorionic; DV, ductus venosus; DV PI, ductus venosus pulsatility index; EDF, end-diastolic flow; FGR, foetal growth restriction; GA, gestational age; iAREDF, intermittent AREDF; MC, monochorionic; REDF, reverse EDF; sFGR, selective FGR; SLPCV, selective laser photocoagulation of placental connecting vessels; RFA, radiofrequency ablation; UAPI, umbilical artery pulsatility index.

* This is the UK-recommended treatment. In Poland, it is not legally possible to terminate a pregnancy or perform selective termination after the end of 22 weeks.

### IUD of one twin

In the case of IUD of one twin, there is a high risk of preterm labour, intracranial changes, neurodevelopmental impairment and death of the co-twin, which are more common in MC than DC pairs due to sharing vascular net. Table 4 presents the prevalence of complications after IUD of a growth-restricted foetus depending on chorionicity.^7^

**Table 4:** The perinatal outcome of the second twin in the case of IUD of sFGR foetus depends on chorionicity.

Perinatal outcome	DC	MC
Second twin IUD	3%	15%
Preterm labour before 32 weeks	54%	68%
Abnormalities in CNS imaging of survivor	16%	34%
Neurodevelopment retardation of survivor	2%	26%

CNS, central nervous system; DC, dichorionic; IUD, intrauterine death; MC, monochorionic; sFGR, selective FGR.

ISUOG recommended (2016) referral to a tertiary centre to perform expert ultrasound with the MCA PSV assessment (screening for anaemia) in a case of IUD of one twin. In preterm pregnancies, the best option is conservative management because of prematurity risks. Ultrasound examination should be performed every 2–4 weeks to evaluate foetal biometry and Doppler (UA, MCA). Foetal neurosonography to exclude intracranial damages is advised after 4–6 weeks following cotwin’s death. In uncomplicated cases, the optimal delivery time is at 34 weeks after a completed course of corticosteroids. In term, pregnancies immediate delivery is the best management.^7^

### ACT in FGR twin pregnancies

Vaz et al.^25^ performed a retrospective analysis of 951 preterm deliveries between 2006 and 2015. ACT administered from 25 weeks and 0 days to 34 weeks and 6 days decrease neonatal morbidity in twins if delivered 7 days before birth.

Similar results were reported in 2018 by Riskin-Mashiah et al.,^26^ who retrospectively analysed 6195 twin infants from a low birth weight database. They showed that ACT decreases neonatal mortality and morbidity in twins complicated by SGA. It should be an option offered in twin pregnancies at risk for preterm delivery at 24–31 weeks.

In 2017, the EPIPAGE-2 prospective cohort study was published. The study population consisted of 750 twin neonates born between 24 and 31 weeks of gestation. It revealed that a single complete course of ACT administered **£**7 days before delivery improved perinatal outcome, and the interval between the treatment and the delivery lasting for over 7 days did not significantly reduce mortality.

In 2019, Martinka et al.,^27^ in their secondary analysis of the twin birth study (2324 twins), reported that in late preterm twins, the benefits of ACT are similar to the effects of therapy in singletons.

On the other hand, there is emerging evidence in the literature that ACT in late preterm pregnancies (after 34 weeks) might not be as beneficial as it was suspected, both in twins and singletons, due to short- and long-term side effects as hypoglycaemia, low birth weight, adulthood diseases such as hypertension, diabetes, polimetabolic syndrome and increased risk of psychosocial distress and psychiatric disorders.^28^

As shown by Viteri et al.^29^ in their study, there are no differences in outcomes in late preterm twins who were administered ACT or not. They suspect that the therapeutic effect might be dose-related and corticosteroid dosage is sub-therapeutic in multiple pregnancies.

## Discussion

Published recommendations and research results do not always agree on the definition, recognition and management of twin FGR depending on chorionicity. This is indeed a double challenge. Applying the Delphi definition of sFGR in DC and MC, twin pregnancies could help standardise research and management internationally. There is a need for further investigations on whether the usage of twin growth charts changes the outcome. However, the singleton charts detect FGR in twin pregnancies better, but at the price of overdiagnosis. The gold standard in diagnosing twin growth restriction, as in singletons, is the differential diagnosis of placental insufficiency, congenital anomalies, chromosomal aberrations or TORCH infection. Growth restriction in twin pregnancies is a double challenge because two foetuses, besides being growth-restricted, may suffer from any of the above. Diagnosis of the aetiology of abnormal growth is the key to planning management. The treatment of sFGR may cause an ethical dilemma due to the co-existence of properly growing and small foetuses in one uterus.

In some cases, *in utero* treatment may cause the smaller twin’s death, but no action might injure the properly growing foetus. A personalised approach is fundamental. In DC twin pregnancies, expectant management is better for the normally growing foetus. Preterm labour may be safe for the sFGR twin, but it may expose the properly growing foetus to complications of prematurity. There is no perfect solution.

Ballabh et al.,^30^ in their study, showed that the half-life of betamethasone was shorter in twin pregnancies than in singletons. Further studies are needed on whether ACT is beneficial in late preterm twins, especially complicated by FGR, and whether the regiment of corticosteroids in twins should be changed in multiple pregnancies due to different pharmacokinetics of the drug.

## Conclusion

Determining the appropriate time for delivery is challenging. In the case of preterm labour, even late preterm, ACT in FGR twin pregnancies is beneficial because it decreases neonatal morbidity. Management of multiple pregnancies complicated by hypotrophy remains a challenge even for an experienced perinatologist.

### Key points

- Published recommendations and research results do not always agree on the definition, recognition and management of twin FGR depending on chorionicity.
- Growth restriction in twin pregnancies is a double challenge because two foetuses besides being growth-restricted may suffer from congenital anomalies, chromosomal aberrations, or TORCH infection, or a combination of aetiologies.
- Determining the appropriate time for delivery is challenging. Preterm labour may be safe for the sFGR twin, but it may expose the properly growing foetus to complications of prematurity.
